# Relationship between histone demethylase LSD family and development and prognosis of gastric cancer

**DOI:** 10.3389/fimmu.2023.1170773

**Published:** 2023-05-03

**Authors:** Liyan Dong, Jiaxing Zhu, Anyi Deng, Junping Wei, Jiawei Li, Xinru Mao, Zhenghu Jia

**Affiliations:** ^1^ Department of General Surgery, General Hospital, Tianjin Medical University, Tianjin, China; ^2^ International Research Center for Precision Medicine, Beroni Group Limited, Sydney, NSW, Australia; ^3^ The Biomedical Translational Research Institute, Jinan University, Guangzhou, China; ^4^ Department of Pathogen Biology, School of Basic Medicine, Tongji Medical College, Huazhong University of Science and Technology, Wuhan, China; ^5^ The First Affiliated Hospital, Biomedical Translational Research Institute and Guangdong Province Key Laboratory of Molecular Immunology and Antibody Engineering, Jinan University, Guangzhou, China; ^6^ Research and Development Center, Guangzhou Purui Biotechnology Co., Ltd, Guangzhou, China

**Keywords:** gastric cancer, histone modification, histone demethylase, protein structure, catalytic mechanism, biological function

## Abstract

**Objective:**

to elucidate the correlation between histone demethylase and gastric cancer

**Research object:**

histone demethylase and gastric cancer

**Results:**

As one of the important regulatory mechanisms in molecular biology and epigenetics, histone modification plays an important role in gastric cancer including downstream gene expression regulation and epigenetics effect. Both histone methyltransferase and histone demethylases are involved in the formation and maintaining different of histone methylation status, which in turn through a variety of vital molecules and signaling pathways involved in the recognition of histone methylation modification caused by the downstream biological process, eventually participate in the regulation of chromatin function, and with a variety of important physiological activities, especially closely related to the occurrence of gastric cancer and embryonic development.

**Conclusion:**

This paper intends to review the research progress in this field from the aspects of histone methylation modification and the protein structure, catalytic mechanism and biological function of the important histone demethylases LSD1 and LSD2, in order to provide the theoretical reference for further understanding and exploration of histone demethylases in development and prognosis of gastric cancer.

## Introduction

**Figure 1:**
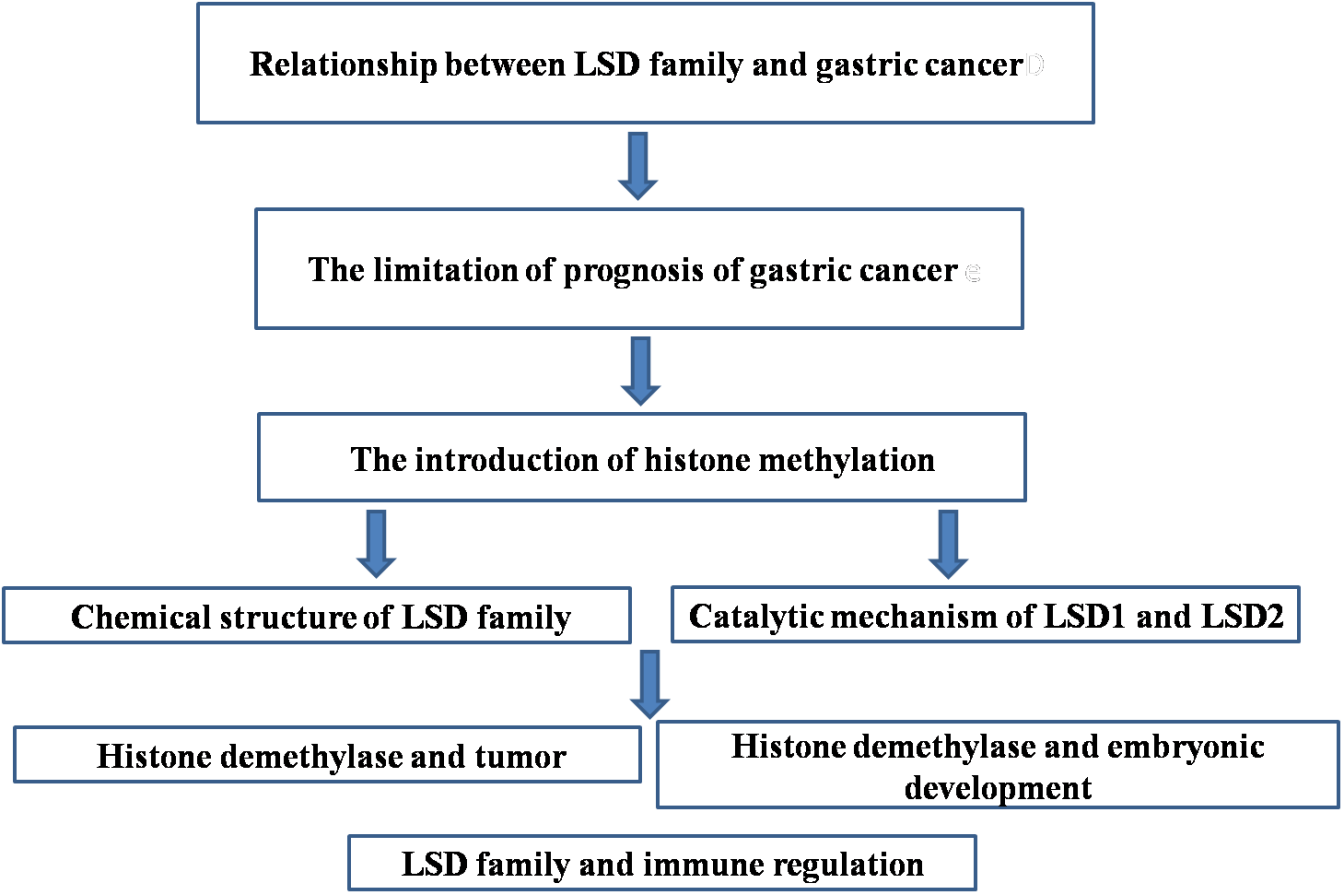
The main contents of this review.

Histone methylation is a reversible and dynamic regulatory process. Methylation and/ordemethylation states are closely related to epigenetic inheritance, transcriptional regulation and maintenance of genome integrity. The histone methylation can directly or indirectly affect a variety of physiological and pathological processes. Histone demethylases are known to include lysine-specific demethylases (LSD) family and Jumonji domain–containing (JMJD) family containing JmjC domain. Studies have found that both of them are closely related to the occurrence of tumors. In this paper, the latest progress of histone demethylase in histone methylation modification and tumor research is summarized, which provides new ideas for the function of histone modification and the study of tumor diagnosis, treatment and prognosis monitoring. In gastric cancer, breast cancer, colon cancer and other common tumors, histone demethylase can change the methylation level of histone or directly act on oncogenes, and also regulate microRNA or transcription factors to promote or inhibit the occurrence and development of tumors and affect the prognosis of tumors. Therefore, in this review, we presented the main contents shown in **Figure 1**.

At present, there are still some problems in our country’s diagnosis of gastric cancer. Although there have been new instruments, new strategies and new techniques applied in the clinical application for the diagnosis of gastric cancer, these new instruments, new strategies and new techniques have not been widely available and the proficiency level of application needs to be improved. Upper abdominal pain is the most common and most overlooked symptom of gastric cancer. Because most gastric cancer often has chronic gastritis and other background diseases. Therefore, the occurrence of upper abdominal pain discomfort, dull pain, fullness and heaviness did not cause attention. Pyloric carcinoma may appear peptic ulcer similar symptoms. In particular, the above symptoms can be temporarily relieved by gastritis or peptic ulcer treatment, and even gastric ulcer can be reduced or temporarily healed. As a result, patients and doctors tend to let their guard down until the disease progresses to an advanced stage. Therefore, when patients have upper abdominal pain and discomfort symptoms, although drug treatment can be relieved, but soon repeated symptoms, immediately should be further examination. Do not misdiagnose gastric cancer by sticking to the so-called “typical symptoms” of “amrhythmic stomach pain” and “growing abdominal pain”. Therefore, it is particularly important to seek reliable molecular targets for gastric cancer diagnosis.

In recent years, epigenetics has become a hot topic in the field of chemistry and biology. With the development of research, scientists have found that epigenetics also has a broad prospect in clinical application. Epigenetics not only promotes the research of life development and gene regulation, but also plays an important role in the prevention, diagnosis and development of new chemical drugs of many diseases such as tumor and inflammation. Among them, histone methylation, as one of the most important epigenetic regulatory mechanisms, is bound to attract extensive attention and research in the world.

## Histone methylation

Histone methylation is an important chemical modification and biological activity *in vivo* (1). In recent years, it has been found that histone methylation is a dynamic and reversible process. Through the interaction of histone methyltransferase and histone demethylase, histone methylation status can be dynamically balanced, the interaction between histone and other important proteins, such as receptors, can be regulated, and the biological processes such as gene transcription and chromosome inactivation can be further regulated (2, 3). Therefore, histone methylation is closely related to human health.

Histone methylation mainly occurs on basic amino acids such as arginine and lysine (4). There are five arginine sites (H3R2,H3R8,H3R17,H3R26 and H4R3) on histones H3 and H4 that can be methylated by PRMT histone methyltransferases (5). Arginine methylation is involved in the activation and inhibition of gene transcription. In addition, six lysine sites (H3K4, H3K9, H3K27, H3K36, H3K79 and H4K20) in histone can be methylated by lysine methyltransferase. The methylation of H3K4, H3K36 and H3K79 is usually related to the activation of gene transcription, while the methylation of H3K9, H3K27 and H4K20 usually regulates the inhibition of gene transcription (1). Histone lysine methyltransferases can be divided into two types: histone methyltransferases with a conserved set catalytic domain of about 110 amino acids and histone methyltransferases without set domain (6). In addition, arginine can be methylated for one time, and for two times symmetrically and unsymmetrically, while lysine can be methylated up to three times. Different methylation states initiate different biological reactions and perform different biological functions *in vivo*.

Histone methylation was generally accepted as an irreversible chemical modification, however in 2004, a revolutionary breakthrough was made in the study of histone methylation, when the first histone demethylase LSD1 was discovered (7). Since then, the view that histone methylation is irreversible has been overturned, with more histone demethylases identified through chemical and biological means. According to the current literature, histone demethylase can be divided into two classes: the first is the JmjC family (Jumonji domain-containing protein), which utilizes divalent iron ions and α-ketoglutarate as coenzymes, demethylated by hydroxylation and the reactants are formaldehyde and succinate (8). The family proteins can demethylate H3K4, H3K9 and H3K36. The second class is histone demethylase, which contains amine oxidase domain and takes flavin adenine dinucleotide (FAD) as coenzyme, and formaldehyde and demethylated lysine as reactants through amino oxidationreaction. It mainly includes two homologous proteins LSD1 and LSD2, which can remove H3K4me1/2 methylation modification and have important biological functions, such as embryonicdevelopment and tumor progression. This paper mainly introduces the histone demethylase of LSD1 and LSD2 amine oxidase family (9).

## Chemical structure of LSD1 and LSD2

LSD1, also known as KIAA0601, p110b, exists in the histone deacetylase (HDAC) complex fromSaccharomyces cerevisiae to human beings, and is a highly conserved functional protein (7). Protein sequence analysis showed that LSD1 contains an N-terminal SWIRM (Swi3p, Rsc8p and Moira) domain, this domain is common to several proteins that interacts with chromosomes contained this domain (10). The Amine Oxidase (AO) domain at the C-terminal is highly similar to FAD dependent Amine Oxidase (11). The protein secondary structure analysis showed that the SWIRM domain contained six α-helices and two β-folds, which can interact with the Amine Oxidase domain. The binding surface area of the two domains is about 1600 nm^2^ (12). In addition, there is an important TOWER domain between the N-terminal SWIRM domain and the C-terminal Amine Oxidase domain. The main function of the TOWER domain is to mediate the interaction between proteins (10). The TOWER domain is formed by two parallel α-helices extending from the Amine Oxidase domain. In the secondary structure of LSD1, the TOWER domain divides the Amine Oxidase domain into two parts (sub-domains), while in the tertiary structure, the two parts of the Amine Oxidase domain are still closely bound to form a complete domain with amine oxidase activity (13). The Amine Oxidase domain consists of two sub-domains, one is FAD bound and the other is substrate bound. The FAD binding sub-domain consists of a certain proportion of α- and β-secondary structures (14). The substrate binding sub-domain consists of six inner β-folds and two peripheral α-helices. These two sub-domains form a large cavity structure, which is confirmed by studying the enzyme activity center of LSD1, the activity center of LSD1 is about 15 angstroms in depth and 25 angstroms in width. The histone substrate can form a stereo stretch conformation when it binds with LSD1 (15).

LSD2 is another important member of the FAD dependent amine oxidase family, although the secondary structure of LSD2 and LSD1 are highly homologous, there are clear differences in chemical structure: the N-terminal of LSD2 contains a unique zinc finger domain with two zinc atoms and a zinc finger-CW domain (16). The zinc finger-CW domain can mediate the localization of LSD2 in the nucleus and participate in the formation of the enzyme activity center of LSD2. In addition, there is no TOWER domain contained in LSD1 in the chemical structure of LSD2. The deletion of the TOWER domain leads to the failure of LSD2 to bind to REST co-repressor (CoREST), and form a complex with HDAC1/2 to regulate gene transcription and expression like LSD1 (17). However, structural chemistry studies showed thatzinc finger-CW domain can regulate gene transcription and expression independently of CoREST and HDAC1-2 (18). In addition, the SWIRM and Amine Oxidase domains of LSD1 and LSD2 have high similarity, but the SWIRM domain of LSD2 contains a unique LOOP region composed of 273-278 amino acid residues (19). Studies showed that the interaction between the SWIRM domain of LSD2 and the substrate was mainly mediated by the LOOP region, as mutations within LOOP region could significantly reduced demethylase activity, indicating that the LOOP region plays an important role in the demethylation by LSD2 (20). In addition, through isothermal titration calorimetry, it was found that the 18-26 amino acid deletion of histone H3 in LSD2 substrate would lead to a significant decrease in the interaction between substrate and LSD2, indicating that the 18-26 amino acid of H3 is particularly important for the binding of LSD2 to substrate (16). Mutations at L20, T22 and R26 of H3 also significantly attenuated the binding of H3 to LSD2, which confirmed that the binding of 18-26 amino acids of H3 to LSD2 is mainly mediated by these three amino acid sites (13).

## Catalytic mechanism of LSD1 and LSD2

Both LSD1 and LSD2 belong to the amine oxidase family. Therefore, LSD1 and LSD2 can cleave the C-N bond of the substrate to form amino group, formaldehyde and hydrogen peroxide. When LSD1 and LSD2 react with the substrate, FAD obtains protons from methylated histone lysine to form FADH2, methylated lysine loses proton to form imine intermediate, FADH2 is oxidized to form FAD and hydrogen peroxide, and imine intermediate is added with water to form amine and formaldehyde (21). When LSD1 and LSD2 catalyze oxidation reaction, there must be a proton on the amine substrate. Therefore, LSD1 and LSD2 can only catalyze 1demethylation of monomethylated and demethylated substrates (3, 22).

Generally, LSD1 can bind to the promoter region of genes and regulate the transcription and expression of genes. Most of the target genes of LSD1 are transcription factors, and other substrate proteins include tumor suppressor gene p53 (23). Different methylation status of p370 at K370 site have different biological functions. For example, K370me1 can inhibit the expression of p53, while K370me2 can promote the binding of p53 and activator 53BP1 (24). In addition, LSD1 can inhibit the interaction between p53 and 53BP1 andreduce the activity of p53 through demethylation of K370 site of p53, thus inhibiting the expression of downstream genes and activation of signaling pathways (25).

Different from the promoter of localization gene, LSD2 is mainly distributed in the coding region of the activated gene. The distribution of LSD2 is similar to that of histone modified H3K36me3, which is involved in maintaining the methylation level of histone H3K4 and H3K9 in gene coding region (26). LSD2 forms a complex with RNA polymerase PolII, transcription elongation factor and other histone modified enzymes (27). The knockout of LSD2 leads to the down regulation of downstream target gene expression. A large number of experimental studies have shown that LSD2 can cooperate with other histone modifying enzymes and transcription elongation factors to regulate transcription extension and the expression of corresponding genes.

## Biological function of LSD1 and LSD2

### Histone demethylase and tumor

In recent years, the relationship between histone demethylase and tumor has become one of the hot spots in clinical research. Clinical studies have shown that, as the core component of Mi-2/nucleosome recombination and deacetylase complex (NuRD), LSD1 is involved in the regulation of a variety of signal pathways related to tumor cell growth, metastasis and invasion, such as E-cadherin snail slug EMT pathway and tumor invasion related TGF-β signaling pathway (28). At the same time, LSD1 can inhibit the invasion and metastasis of breast cancer cells. It can regulate the proliferation of breast cancer cells and epithelial mesenchymal transition by reverse regulation of TGF-β1 signaling pathway (29). In addition, LSD1 plays an essential role in Snail/Slug (transcription factor) mediated EMT. The deletion of LSD1 will lead to the failure of transcription factors to inhibit the expression of E-cadherin, and Snail/Slug will not be able to independently inhibit the expression of E-cadherin gene (30). The inhibition of E-cadherin on E-cadherin promoter is realized by epigenetic regulation of histone in E-cadherin promoter region by LSD1. In addition, LSD2 is also highly expressed inmany tumor cells. For example, in prostate cancer cells, LSD2 and FHL2 can specifically bind to androgen receptor (AR) to activate the expression of androgen related target genes (19).

### Histone demethylase and embryonic development

Histone demethylase plays an important role in embryonic development. Knockout of LSD1 in Drosophila embryos will lead to the disorder of H3K4 methylation and the corresponding downstream gene abnormalities, which will eventually lead to the death of embryos (25). In addition, some studies have found that during the reproduction of nematodes, the histone modification map will be partially removed, which can not be inherited by the offspring, and the mutation of LSD1 will lead to dysfunction of the function of removing histone modification map of fertilized eggs and affect the normal development of offspring (24).

In the process of gametogenesis, the difference of homologous chromosome methylation between male and female parents will regulate the differential expression of imprinted genes. Theresearch results of Han et al. showed that high expression of LSD2 in oocytes is crucial for *de novo* methylation of imprinted genes in oocytes (23); LSD2 deficiency can significantly increase the methylation level of H3K4 in oocytes, and the normal methylation level of most imprinted genes cannot be realized. Additionally, LSD2 deficiency leads to embryonic death prior to the embryo originated from oocyte will die in second trimester of pregnancy (3).

## LSD1 and LSD2 and immune regulation

Previous studies have shown that LSD1 and LSD2 play an important role in the regulation of autophagy and differentiation of stem cells, hematopoietic cell differentiation, and tumor epithelial mesenchymal transformation. LSD1 and LSD2 are essential for maintaining cell dryness (31). After LSD1/LSD2 knockout, the differentiation balance of mouse embryonic stem cells is impaired, the cell cycle process is disturbed, cell death increases, and serious growth disorders or stillbirth occur. LSD1/LSD2 plays multiple functions in the hematopoietic system, such as regulating the self-renewal of hematopoietic stem cells, balancing the differentiation of red blood cells and myeloid cells, and maintaining plasma cell dryness (32). Deletion of LSD1/LSD2 can increase the level of hematopoietic stem cells and progenitor cell gene H3K4me1/2, and affect the differentiation of early hematopoietic stem cells, late granulocytes, and red blood cells (33). LSD1/LSD2 knockout in mice results in severe hematopoietic stem cell differentiation and mature hemocytosis defects, with a large decrease in hemocytosis (34). Epithelial mesenchymal transformation refers to the acquisition of mesenchymal cell phenotype by epithelial cells, which is necessary for tumor cell invasion and metastasis. LSD1/LSD2 can promote tumor invasion and metastasis by inhibiting the transcription of E-cadherin, a key EMT molecule. It can also inhibit the EMT process of tumor by inhibiting the expression of TGF-β (35). Clinically, high expression of LSD1/LSD2 is associated with poor prognosis of various tumors. Targeting LSD1/LSD2 has become an important direction of tumor therapy, especially acute myeloid leukemia and breast cancer. Currently, LSD1/LSD2 inhibitors ORY-1001 and GSK-287552 have entered clinical trials for the treatment of acute myeloid leukemia and gastric cancer (36).

Recent studies have found that LSD1/LSD2 also plays an important role in immune regulation. LSD1/LSD2 can affect the differentiation of monocytes into macrophages and inhibit THP1 differentiation into macrophages (37). In acute myeloid leukemia cells, inhibition of LSD1/LSD2 induces stem cell-like leukemia cells to differentiate into macrophages and express CD14 and CD11b on their surfaces (38). In melanoma cells, inhibition of LSD1/LSD2 enhances tumor immunogenicity and promotes T cell immunity. LSD1/LSD2 can also be synthesized by Bcl-6 with B cells (39). Otherwise, in macrophages, LSD1/LSD2 mediates demethylation of p65 promoter region, which can improve the stability of p65 and maintain the inflammatory response during the duration of inflammation (40). In gastric cancer mice, LSD1/LSD2 inhibitor pargyline can inhibit EMT and promote the immune response of M1 macrophages in conjunction with chemotherapy for gastric cancer (41).

Therefore, the main contributions of LSD family can activate and inhibit the transcription of corresponding genes, and is closely related to the occurrence and development of many diseases, such as gastric cancer, embryonic development and immune regulation.

## Conclusion and prospect

Although the research into histone demethylase of LSD1 and LSD2 amine oxidase family is a recent hotspot and development, it has progressed rapidly, benefitting from contributions from studies on epigenetics and protein structural chemistry, especially the discovery of chemical structure and biological function of LSD1 and LSD2. These findings lay a solid foundation for all future histone demethylase research. We believe that more and more chemical structures and biological functions of histone demethylase and their relationship with major diseases will be discovered through the continuous improvement of research methods such as cell level and animal models, and the interdisciplinary research of chemistry, biology and information technology, which provides the possibility for developing new chemical drug targets and proposing new clinical treatment strategies.

## Author contributions

ZJ designed the idea of review; LD and JZ performed the collection and collation of references; JW, JL, LD and XM wrote the paper. All authors contributed to the article and approved the submitted version.
